# Teaching in a lockdown: The impact of COVID-19 on teachers' capacity to teach across different school types in Nigeria

**DOI:** 10.1016/j.heliyon.2023.e13781

**Published:** 2023-02-15

**Authors:** Seun Bunmi Adebayo, Gbenga Quadri, Samuel Igah, Obiageri Bridget Azubuike

**Affiliations:** a University of Galway, Ireland; b TEP Centre, Nigeria; c University of Bristol, United Kingdom

**Keywords:** Teachers, Teaching and learning, COVID-19, Education in emergency, School type, Nigeria

## Abstract

Using the Capability Approach as a theoretical lens, this study employed mixed methods to examine teachers' capacity to deliver teaching across school types during closures caused by the COVID-19 pandemic in Nigeria. The data analysed for this study was collected using an online survey and semi-structured interviews via phone with 1901 respondents, including teachers. This study investigated the support and resources available to teachers to contribute to quality teaching remotely using online learning platforms. Our findings showed that even with the expectation for teachers to continue teaching in a pandemic, many teachers in Nigeria lacked the pedagogical competencies and resources to deliver teaching remotely or virtually. We, therefore, recommend an urgent need for ministries of education to prioritise addressing challenges confronting teachers and equip them with the required pedagogical competencies and resources to deliver online learning even during a humanitarian emergency.

## 1 Introduction

Humanitarian emergencies and crises have been identified by research to affect teachers' work, particularly in the understanding of their professional agency within education systems situated in the above context [1,2]. This is problematic given that teachers are critical stakeholders in fulfilling educational reforms and goals such as the Sustainable Development Goal (SDG) 4: *Ensure inclusive and equitable quality education and promote lifelong learning opportunities for all* by 2030 [3]. While teachers' work mainly includes teaching and implementing the national curriculum [4], the outbreak of the Corona Virus Disease (COVID-19) disrupted education systems globally, and more than a billion learners were affected as a result of the pandemic [5]. Teachers were also affected by the pandemic, and within short notice, teachers were expected to take on new responsibilities in ensuring that students could continue learning during the lockdown periods of the COVID-19 pandemic [6]. Furthermore, COVID-19 pandemic has highlighted inequalities inherent in many education systems, particularly in developing countries [7]. The pandemic amplified inequalities, such as the lack of access to equitable and quality education for marginalised learners [8]. With learning institutions closed for months worldwide, many learners were denied education, especially if they could not access online learning platforms [[9], [10], [11]].

Furthermore, teachers in developing countries, particularly in Sub-Saharan Africa (SSA), were confronted with challenges that affected their work. Many faced challenges such as lack of institutional support, poor wages, low motivation, and inadequate training and resources [1,4], and teachers in Nigeria were not left out. In this study, we argue that there is a need to understand the impact of COVID-19 on teachers' capacity to teach during the school closures in Nigeria caused by the pandemic. This study is significant because the world still grapples with understanding the impact of COVID-19 on education globally. Additionally, there is limited research in Nigeria on how school closures caused by the pandemic affected teachers' capacity to teach across different school types – public and private. Therefore, our study responds to this gap in knowledge by identifying the challenges of quality and equitable access to education technology for online learning, especially for marginalised learners. Our rationale for examining differences between school types is based on existing inequalities between Nigeria's private and public education sectors. Research shows that teachers’ ability to provide high-quality instruction and learning is common in private schools compared to public. Also, compared to public schools, most private schools tend to increase teachers’ capability for quality teaching by providing the right support and resources [[12], [13], [14]].

Furthermore, this study highlights how teachers were caught between a lack of pedagogical competencies for online learning and dire work conditions for delivering learning in a pandemic. Findings from this study, in particular, will help policymakers and educational stakeholders to redefine teacher support, address teacher challenges and, ultimately, teacher education for better teaching and learning [15], especially during periods of humanitarian emergencies and crises.

In Nigeria, after the closure of schools on 19 March 2020 by the federal government, both private education service providers and state governments introduced online learning platforms to facilitate students' learning [16]. However, according to a report by The Education Partnership Centre [TEP Centre] (2020) [6], the lack of resources, teacher well-being and pedagogical support for teachers to deliver lessons online were crucial challenges to teachers' work during school closures in Nigeria. Therefore, our paper investigates the following research questions: *A. What was the impact of COVID-19 on teachers' capacity to teach during school closures in Nigeria? B. Are there differences between private and public schools on teachers’ capacity to continue teaching during school closures in Nigeria?*

We answer the above research questions by exploring the following themes from this study, student engagement, teachers’ capacity across different school types and the challenges of remote teaching.

In light of the above, we explore the impact of COVID-19 on teachers' capacity to teach during school closures in Nigeria. The paper starts with the Capability Approach adopted as the theoretical framework for this study. After that, a review of teachers and teaching across different school types in Nigeria is presented. The paper continues with the research methodology adopted for the study. Thereafter, findings and discussions are presented. The paper concludes with the implications and limitations of the study.

## 2 Theoretical framework: capability approach

Capability Approach (CA) by Amartya Sen lays a foundation for the importance of social and economic empowerment to members of a society for sustainable human development [17,18]. According to the CA school of thought, equal opportunities can be guaranteed in a society when equal capabilities are allowed for every member of the society [19]. In other words, CA empowers people with the resources they value to be whom they want to be. The CA aligns with the principles of social justice by advocating for equal opportunities for all [19].

Within the context of education studies, CA presents education as a tool of empowerment that will lead to sustainable human development through the equal redistribution of resources to all [20]. The values of education under the CA include making members of a society active participants in decision-making processes, empowering them with the capabilities to thrive and advocating for equal distribution of resources [20,21]. According to Tao (2013) [22], CA can be useful in understanding teachers' behaviour and quality. Notably in examining the quality of teacher education and challenges and their impact on teachers performing their professional obligations. Tao (2013) [22], in her study on teachers' behaviours in Tanzania, strongly opines that the instrumentality of teachers contributing to quality education cannot be overemphasised. “However, the issue of teachers' capability deprivation has yet to be thoroughly investigated, particularly in regard to how this may manifest itself in particular behaviours such as de-motivation, absenteeism, and lack of preparation” [4,22].

Drawing ideas from the CA, we first seek to understand the state of the teaching profession, mainly how teachers across different school types in Nigeria continued teaching during the COVID-19 pandemic. We further investigate the support and resources available for teachers to continue contributing to quality education using online learning platforms with the closure of schools due to the global pandemic. We argue that in line with the tenets of CA, teachers need to have the necessary pedagogical skills, resources and support to effectively teach during a lockdown. The COVID-19 pandemic has highlighted the critical role of teachers during this time [5]. No doubt the COVID-19 pandemic has profoundly impacted the world of education, with countries facing numerous challenges in the transition to online learning [23]. Firstly, the rapid shift to remote education required a significant adjustment in how teachers and students approach learning. Many educators lacked the skills or resources to facilitate effective online instruction. Additionally, many countries such as Kenya, China, Taiwan, and Kazakhstan were not adequately prepared to provide all students with access to technology and the internet, compounding online learning challenges [[24], [25], [26], [27]].

Moreover, the COVID-19 crisis has also revealed significant disparities in access to technology and resources. Students from low-income families or rural areas were often left behind in the transition to online learning. This has further exacerbated existing educational inequalities, creating a digital divide between students with access to technology and those without access [24,28]. We, therefore, posit that it is crucial to examine the impact of the pandemic on teachers and teaching in Nigeria from the CA analytical lens. Furthermore, the extent to which teachers' work is affected during the pandemic might be proportional to the current capacity of teachers to deliver teaching via online learning channels. Therefore, the CA framework allows us to not just ‘blame’ teachers for the lack of effective teaching during the pandemic but to interrogate the impact of COVID-19 on teachers' capacity to deliver expected learning to their students during school closures.

## 3 Literature review

### 3.1 Teachers and teaching across school types in Nigeria

Research has established that teachers are vital in providing quality education to students, particularly in humanitarian emergencies, as they are responsible for implementing the national curriculum within classrooms [2,29]. Therefore, it is expedient to understand the relationship between teachers' capacity and their adaptation to teaching during the COVID-19 pandemic. Particularly when drafting education policies for quality teaching and learning for health emergencies like the COVID-19 pandemic and the future of learning. Teachers in low-middle countries are often less involved in education decision-making processes, including policies that affect teachers' work and the teaching profession [2,30]. The result of not taking cognizant of teachers' experiences and their first-hand experiences of classroom realities have led to unsuccessful outcomes of education reforms [2,29,31]. According to Fafunwa (1969) [32], teachers are trained subject professionals possessing the necessary qualifications or academic degrees to teach within a learning institution. For this article, we present that teachers in the context of the SDG 4 are qualified professionals who work in both formal and informal educational settings [3].

In Nigeria, teachers are seen as role models and counsellors to their students, keepers of culture, and intermediaries between schools and society [33]. No doubt that this is the case with teachers in many countries [29], teachers in Nigeria have been fundamental to the country's development at all levels of society [34]. However, according to Osai (2016) [35], teachers in Nigeria are mostly unmotivated due to low salaries, poor working conditions, low social status and lack of adequate training. These challenges facing teachers in Nigeria have contributed to poor teaching and learning outcomes in schools [2]. Furthermore, Osai (2016) [35] argues that teachers largely determine the type of students produced for sustainable societal development. In other words, the quality of teachers in schools determines the quality of learners. Therefore, teachers' capacity and welfare must be prioritised within education systems to contribute to quality teaching and learning outcomes [2,22,30].

Recent studies show that not all Nigerian teachers were equipped to deliver quality education during the COVID-19 lockdown for many interconnected reasons, including training-related issues, infrastructural limitations, and low remunerations [6,9]. According to Gimba (2012) [36], no country can build a strong and effective educational system without the continuous appraisal and improvement of its teachers' capacity and training programme since teachers remain the pillars and bedrock of the system. According to Omede (2015) [14], in Nigeria, the capacity of teachers to deliver quality teaching and learning is primarily determined by the type of school - either public or private, in which they work. Additionally, quality teaching is observed to be more available in private schools because teachers are empowered with needed resources to achieve learning goals [14].

For this paper, teachers' capacity is defined as the ability of teachers to perform their functions effectively, efficiently, and sustainably with required resources being made available to them [30]. Further, Glewe (2002) [13] argues that teachers' capacity is generally affected by the school type, as most private schools tend to improve the capacity of their teachers more than public schools. However, Archibong and Okon (2009) [12] present that the ratio of trained public school teachers is higher compared to private schools. In other words, there is an ongoing challenge with teacher qualifications in private schools; many teachers within the private education sector often do not possess the right teaching qualification required by the Ministry of Education [12]. While in the literature, school type has been classified as public or private, rural or urban, same-sex, or mixed-sex [[12], [13], [14]]. In this research study, we define school type in relation to its ownership. In Nigeria, schools owned by the federal or state governments are known as public schools. While other schools are classified as private schools, and this type of schools have private ownership; that is, they are owned by individuals or a group of individuals [14]. In recent years, the absence of adequate infrastructure and facilities in public schools has hampered teachers' performance in public schools [37]. According to Omede (2015) [14], parents in Nigeria prefer to enrol their children in private schools over public schools because private schools have better facilities like modern laboratories, libraries, classroom furniture, and recreational equipment than public schools.

#### 3.1.1 Teaching during the COVID-19 pandemic

More globally, the impact of COVID-19 on education resulted in 1.6 billion learners missing out on schooling during the peak of the pandemic [38]. Moreover, evidence from education systems across many countries shows that the pandemic led to increasing educational inequalities, deteriorating students' and teachers' well-being. Also, the low educational technology adoption in developing countries contributed to growing learning loss among marginalised groups [[37], [38], [39]].

Online teaching is what many schools and teachers employ in lesson delivery to their students during school closures caused by the COVID-19 pandemic [6]. The school closures lasted for six months in Nigeria between March and September 2020 [40], and many teachers in Nigeria identified that a lack of resources and online pedagogical knowledge led to rogue teaching during the pandemic [6,41]. The above reality has amplified the need to ensure that teachers who are saddled with the critical responsibilities of delivering education to students should be positioned appropriately for 21st-century learning [11,42]. We posit that teachers need to be equipped with online pedagogical skills to deliver online learning to students, which aligns with the arguments of the CA. Therefore, teachers need to be adequately trained and empowered with adequate resources to implement online learning and teaching processes effectively.

## 4 Methodology

### 4.1 Research design

The data analysed in this research study is from The Education Partnership (TEP) Centre and the Nigerian Economic Summit Group (NESG)^1^ Education innovation survey conducted between April and May 2020. The study period covers the first two months of school closures due to the COVID-19 pandemic in Nigeria.

### 4.2 Participants

In this study, we focus on data gathered through teachers' responses with a sample size of 439 teachers across 31 out of 36 states in Nigeria. The average age of the teachers in the survey was 37.76 years with a standard deviation of 10.60 years. There are more female respondents at 56% of the sample than male teachers (44%) in the sample. Furthermore, 28% of the teachers had been teaching for less than five years, while 72% of the sample had more than five years of teaching experience. 54% of the teachers were public school teachers, 46% taught in private schools, and the majority of the teachers taught at the upper secondary school level. Table A1 in the appendix provides a description of the sample of teachers in the study.

### 4.3 Sampling approach

The survey employed both purposive sampling (approaching the respondents fit for the survey) and a snowball sampling approach (asking survey respondents to recruit their acquaintances eligible to complete the survey). This sampling approach is in line with the position of Best and Khan (2007) [43] that research participants are selected with the expectation that they can provide valuable information in answering research questions.

### 4.4 Data collection

The data was collected online and through semi-structured interviews administered via phone to 1901 respondents. The research respondents included teachers. The respondents resided in 35 of the 36 states in Nigeria, including the Federal Capital Territory. The survey was administered online via Google Forms and telephone interviews. 53% of the respondents completed the survey online, while the remaining respondents were reached through phone calls. The data is both quantitative and qualitative because the survey questions were both closed-ended questions and open-ended questions that allowed in-depth responses from the survey respondents. Data validity and verification exercises were conducted through a random selection of 10% (87) of the respondents who participated in the phone interview survey, and 86 of the respondents confirmed that they were interviewed through telephone calls.

### 4.5 Ethical considerations

The data was collected anonymously, and no ethical concerns were raised regarding the information collected through the survey as they focused on access to learning and teaching capacity during the COVID-19 pandemic and school closures. Furthermore, the data was collected by TEP Centre, a registered organisation in Nigeria with many years of data collection and education partnerships with private and public sectors in Nigeria. The confidential use of the data collected was respected, and informed consent was obtained from all the participants before the study commenced.

### 4.6 Data analysis

As a result of the diverse nature of the qualitative and quantitative data collected for this study, we employed a mixed-methods approach in the data analysis. According to Almalki (2016) [44], mixed methods research has greater potential to provide depth as well as breadth for the research in ways that a singular approach may not provide. For analysis of the quantitative data, we employed descriptive analysis (using tables and graphs), and Chi-Square tests of independence (at 95% level of statistical significance). The Chi-Square test of independence is used to determine whether two categorical variables in a single sample are independent from or associated with one another [45]. The Chi-square test is appropriate for the quantitative analysis because the teachers are defined in two categories – private and public schools and the outcomes of interest (such as student engagement and effectiveness of virtual learning platforms) are also defined in categories. The null hypothesis is that the variables of interest are independent, while the alternative hypothesis states that the variables of interest are associated. A significant result of the Chi-square test will lead to a rejection of the null hypothesis of independence [45].

For the qualitative analysis we use a reflexive thematic approach with direct quotations [46,47]. With thematic analysis, we were able to generate relevant themes in providing answers to our research questions. We followed the six steps of reflexive thematic analysis according to Braun and Clarke (2006, 2020) [[46], [47]]. Firstly, we familiarised ourselves with the qualitative data, then the research team performed open coding; thereafter, we generated initial themes and then reviewed the themes. We concluded the process by refining the themes and then presented them as part of the findings of our study. Since a relatively huge dataset was used for this research study, thematic analysis allowed us as researchers to critically explore the usefulness of the data and what is relevant to helping us achieve our identified research objectives [46,47]. The use of a mixed-methods approach to our data analysis conforms to the principles of the CA adopted as an analytical lens for this research paper.

## 5 Findings

In this paper, we explore the impact of COVID-19 on teachers' capacity to teach during school closures and across different school types in Nigeria. Major themes from our analysis include teachers' engagement with students across different school types during school closure, teachers' capacity to teach during a lockdown, and the challenges of teaching remotely during the COVID-19 pandemic.

### 5.1 Teachers' engagement with students across different schools during school closure

Teachers in this study reported that they engaged their students through different means, including social media, radio and television programmes. 41% reported that their students were learning through virtual platforms, 39% reported that their students were learning via radio and television programmes, and 28% reported that their students were not actively learning. We further examine the responses of teachers by the type of school they were teaching in. We find a significant association between school type and whether teachers reported that their students were learning through virtual learning platforms or radio and television programmes.

From Table 1 above, teachers teaching in private schools where more likely to report that their students were learning via virtual learning platforms (54%) than teachers who taught in public schools (29%), and the association was statistically significant *X*^*2*^ (1) = 30.03, *p* < .001. Teachers who taught in public schools were more likely to report that their students were learning through radio and television programmes (54%) than those who taught in private schools (22%), similarly this association was statistically significant *X*^*2*^ (1) = 46.39, *p* < .001. Private school teachers were also more likely to report that they offered support to parents home-schooling their children (21%) compared to public school teachers (10%) the association was statistically significant *X*^*2*^ (1) = 10.63, *p* < .001.

**Table 1:** How teachers are keeping students engaged during the pandemic.

Responses	Private school		Public school		Private vs Public∗
	%	Freq	%	Freq	(P-Value)
Teaching a cohort of pupils/students in a designated location?	2.97	6	2.53	6	0.779
Teaching pupils in their homes?	14.85	30	9.28	22	0.072
Students are not actively learning?	25.74	52	29.54	70	0.38
Students learn via radio and television programmes?	21.78	44	53.59	127	<.001
Supporting parents in home-schooling children?	20.79	42	9.70	23	<.001
Students learn via virtual platforms?	54.46	110	28.69	68	<.001

∗ Chi-square test for association at 95% statistical significance level (Sig. p < .05). Reporting for yes responses only. N = 439, Private school = 202 and Public school = 237.

In the states where radio and television programmes were the alternative forms of learning provided by the state government, all children from both public and private schools could access the programmes, so while private school students learned via online programmes provided by their schools, they could also access publicly available content on television and radio.

### 5.2 Teacher's capacity to teach during a lockdown and across different school types

With the closure of schools due to the pandemic, teachers were asked if they were teaching their students using virtual learning platforms. 52% of the respondents reported teaching their students using virtual learning platforms. When asked to rate the effectiveness of the virtual learning platforms they were deploying for teaching. 34% reported that the platforms were good, 31% gave a neutral response (i.e., neither good nor poor), 22% reported that the platforms were very good, and 9% and 4% reported that the platforms were poor and very poor, respectively. We then disaggregate the results by school type and report the findings in Table 2 below.

**Table 2:** Teachers' rating of the effectiveness of virtual learning platforms by school type.

Rating	Private school		Public school		Total
	%	Freq	%	Freq	N
Very poor	55.56	5	44.44	4	9
poor	28.57	6	71.43	15	21
Neutral	50	36	50	36	72
Good	58.97	46	41.03	32	78
Very good	69.23	36	30.77	16	52
Total	**55.6**	**129**	**44.4**	**103**	**232**

Reporting for only teachers that used virtual learning platforms. N = 232, Private school = 129 and Public school = 103.

From Table 2 above, private school teachers gave more positive (good or very good) ratings of the virtual learning platforms they were utilising, and teachers in public schools gave more negative (poor/very poor) ratings. In further analysis, we found a statistically significant association between school type and the ratings of the effectiveness of the learning platform *X*^*2*^ (4) = 11.40, *p* = .022. When asked why the poor ratings were given, teachers cited infrastructural challenges such as data costs, internet connectivity and electricity. They also cited pedagogical issues with remote teaching, such as non-familiarity with the platform being used, inability to assess students remotely and a lack of interpersonal relationship between students and teachers. One female teacher in a private school gave the virtual learning platform through radio and television a poor rating for the reasons cited below:

Because learning at home does not give room for assessing whether the child understands what is being taught or not. It also has no room for the pupils to ask questions.

Another female public school teacher utilising WhatsApp reported that:

(…) many find it difficult to access the internet. Many parents cannot afford browsing phones. And those on WhatsApp are finding it a little difficult to access it due to poor network…

Private school teachers were more likely to give a poor rating due to pedagogical challenges, as in the case of the private school teacher cited above, and public school teachers were more likely to give the platforms poor ratings as a result of access and infrastructural challenges faced by students in accessing learning remotely during school closure.

Furthermore, teachers were asked about the provision of resources by their employers to continue teaching during the lockdown (Table 3). In further analysis of the association between school type and provision of resources to continue teaching, we found that although private school teachers were more likely to report that their employers provided them resources to continue teaching, the association was not statistically significant *X*^*2*^ (1) = 3.61, *p* = .057.

**Table 3:** Has your employer provided the resources for you to support your students' learning.

Support provided	Private school		Public school		Total
	%	Freq	%	Freq	N
**No**	49.29	69	50.71	71	140
**Yes**	60.98	75	39.02	48	123
**Total**	**54.75**	**144**	**45.25**	**119**	**263**

Reporting for only teachers that received support from employers. N = 263, Private school = 144 and Public school = 119.

Teachers were asked what resources their schools provided to support the continuation of learning. Private school teachers reported more that they were given internet data, digital devices and financial support to continue teaching during school closure.

One private school female teacher reported:

My employer provides me with data and laptop. This is what we have been using before the lockdown, so we just continued. She constantly top up the data to enable us research on other creative ways to teach children from our homes.

A male public school teacher, on the other hand, reported that his employer provided:

Timing for the various learning programmes on local radio and television stations, as well as making relevant free learning websites available for interested students, teachers and parents.

Another male public school teacher reported that:

Development of digiclass^2^ on radio and TV. Drawing of time table. Provision of experienced and qualified teachers to handle each subject.

From the findings above, the differences in teaching resources and capacity during the lockdown are apparent in that private school teachers were provided tools that empowered them to continue teaching via virtual learning platforms. They utilised tools such as Google Classroom and Zoom, while for public schools, a few (qualified) teachers were supported to teach through television and radio.

**Fig. 1:**
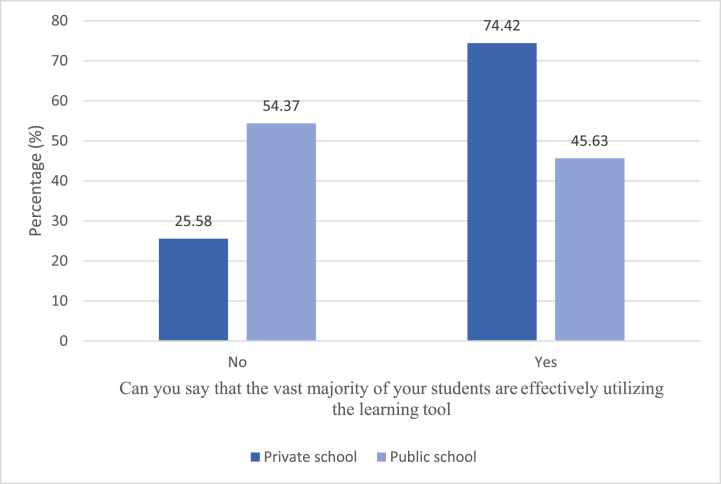
Teachers report on students' effective utilisation of learning tool.

From the below Fig. 1, teachers were asked if their students effectively utilised the learning tools they reported using in teaching their students. We find a statistically significant association between the type of school of the teacher and if the teacher reported that their students were effectively utilising the learning tools *X*^*2*^ (1) = 20.07, *p* < .001. Teachers teaching at public schools were more likely to report that their students were not effectively utilising the learning tool compared to teachers in private schools.

Teachers were asked why the students were not effectively utilising the tools. Teachers reported that access to digital tools, internet connectivity and electricity, in some cases, were reasons their students could not effectively utilise the learning tools.

According to one public school teacher:

No access to the learning tools, they are in remote areas so trying to encourage radio translation of classes in their local dialects as means of passing some form of knowledge to these students while out of school.

Another public school teacher reported:

The issue is because most of their parents reside in places without a network. And those living within a network area find it difficult to purchase android phones, as they would rather buy food with the money to cater for the basic needs of the family.

Teachers teaching in low-resourced environments faced both challenges from inadequate provisions for them to teach effectively and challenges from the students' side when their parents could not provide the needed resources for learning.

When asked if their students had adequate access to resources they needed to learn remotely, 77% of teachers in public schools reported that their students did not have adequate access to the resources they needed to learn, compared to 45% in private schools. Similarly, 71% of teachers in private schools reported that their students had access to the learning resource they needed compared to 24% in public schools, which was found to be statistically significant.

### 5.3 Challenges of teaching remotely during the COVID-19 pandemic across different school types

**Fig. 2:**
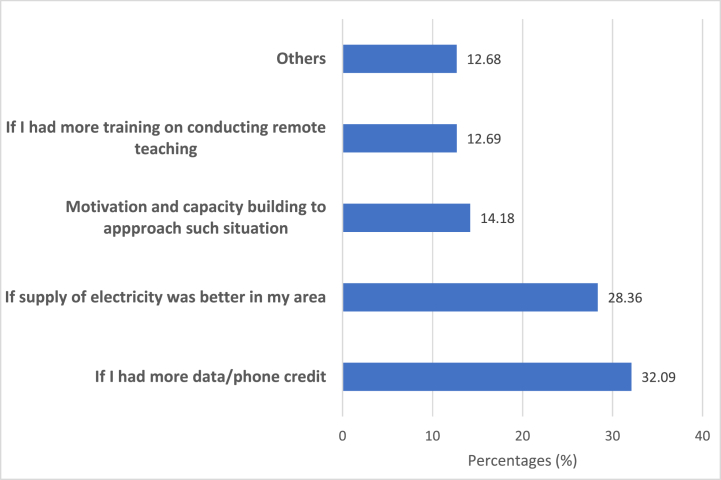
What would make teaching remotely easier?.

Teachers were asked; how easy has it been supporting your students learning virtually in this period? About 49% reported that it was easy (i.e. fairly easy or easy), while the other 51% reported that it was difficult (i.e. difficult or fairly difficult). When we analysed these differences across school types, we found no statistically significant differences between school types and teachers' reports of the ease of teaching remotely. Teachers who reported difficulties teaching remotely were asked the following question; *If it has been difficult or fairly difficult to* support *your students' learning at this time, what would make it easier?* 32% reported that having more internet data or phone credit would make it easier to teach remotely, implying that remote teaching resources like internet data were the leading cause of difficulty for them. Furthermore, 28% reported that better electricity would make remote teaching easier, 14% reported that motivation and capacity building to approach teaching in a lockdown would make it easier. See Fig. 2 below for a graphical representation of the above finding.

Furthermore, when asked what support teachers needed to continue teaching their students remotely/virtually, the themes that emerged from teachers' responses included infrastructural, financial, parental, community and pedagogical supports.

We present below some qualitative data on supporting teachers to deliver teaching during a pandemic in Nigeria:

*Need support in getting relevant training on the adequate tools for remote teaching and learning, Also sensitising all stakeholders on their crucial roles in making this possible for all learners … -* Public school teacher.

*Moral support in the form of encouragement and feedback from the students. Without feedback, there can be no good knowledge of results, and without a good knowledge of results, there can be no systematic improvement in learning*. - Public school teacher.

*I need data to teach those on WhatsApp and credit to be calling my pupils who are not on WhatsApp and doing the teaching. I would love to have a laptop, too for effective teaching also*. - Public school teacher.

*Support is to enlighten me on ways I could teach my pupils online and make their parents respond well.* – Private school teacher.

*An employer should continue to provide data to use. Parents should also make their children available and ready during the lesson period online.* – Private school teacher.

The responses from teachers show that both private and public school teachers faced challenges implementing teaching during the lockdown, and although there were no systematic differences between their reports of difficulties in implementing remote learning, the themes emerging from their report of support show some differences as cited from the above direct quotations of teachers in private and public schools respectively.

## 6 Discussion

Our findings in this research study revealed that teachers used various tools to teach their students during the pandemic and across different school types. Data showed that 65% of teachers in the study used Zoom, 56% employed social media messaging applications such as WhatsApp, Facebook, and Telegram. 48% taught their students via a number of Android and IOS applications, while 35% reported teaching via television and 25% reported via radio. Furthermore, we found that teachers in private schools were more engaged in teaching remotely during the lockdown and doing so through platforms such as Zoom, and other virtual learning platforms compared to teachers in public schools. Other studies have observed that students from public schools were more likely to learn via radio and television programmes during the pandemic [9,11,48]. The above finding is instructive because we argue that educational inequalities have been amplified in Nigeria as a result of the COVID-19 pandemic [11,49], as school closure sorted students of differing school types into different forms of learning that require different resources but also lead to the development of different skill sets.

We also found that teaching and learning differed across students depending on their school. As fewer teachers were needed to teach via radio and television, only a handful of public school teachers were effectively engaged with teaching virtually during the lockdown. For other public school teachers who adopted mobile phone applications such as WhatsApp to teach their students, they faced challenges such as internet connectivity and internet data accessibility on the part of their students. All of these raise concerns about teachers' capacity to teach their students and the equality of access to education and digital learning during the lockdown [11]. Therefore, from this finding, we posit that the capacity of teachers to teach during school closures was influenced by the digital infrastructure gap that has deepened educational inequalities within Nigeria's education sector [9,11]. The inability of the public education system to adequately cater for the digital needs of all students during the school closures as a result of the pandemic calls for an urgent need for governments to sufficiently fund the development of public schools to ensure that all learners have access to equitable and quality education even in a humanitarian emergency [3,11] Adequate provision of educational technology for all learners will lead to improved teaching and learning outcomes [50].

Using the CA as an analytical framework, we move beyond the ‘blame game’ that blames teachers as the main cause of students' inability to learn during a learning crisis. Based on our findings, in both public and private schools, teachers were largely unprepared pedagogically when they were expected to continue teaching via digital learning platforms within a short period. Therefore, we argue that teachers' capacity to teach is a function of the provisions from their employers [30,31] and the provisions that parents make to ensure their children can learn [51]. This finding also agrees with Tao (2013, p. 4) [22] that teachers manifest certain behaviour due to their ‘own capability deprivation.'

Research has confirmed the need to adequately equip teachers and schools with the resources to deliver quality teaching and learning outputs [2,22,30,31]. Additionally, our findings clearly demonstrate that school type determines the resources provided for teachers to teach their students [14], but it is also linked to the resources that parents can provide for their children's learning, which influences the teacher's capacity to teach the child. If a teacher has the needed tools, but the students cannot access the tools needed for learning, then learning cannot happen [11,51].

Based on the above, we, therefore, posit that teachers' capability to deliver quality teaching can be improved when their needs, such as infrastructural and financial support, parental and community support and pedagogical support, are provided [9,11].

## 7 Conclusions, implications and limitations

With the closure of schools in Nigeria in April 2020, teaching moved from face-to-face physical teaching and learning to remote or virtual learning because of the pandemic. The inequalities arose in areas where students could not learn outside of a physical classroom either because they had no access to virtual learning tools or where teachers could not teach. After all, they could not do so remotely. Therefore, the closure of schools exacerbated inequalities inherent in many education systems worldwide, including Nigeria.

The strength of this study is that the results raise the need for adequate support for the students who experienced a learning slide during the lockdown due to their inability to learn. The inequalities in learning caused by the pandemic could continue as learning gaps even with the resumption of schools. If the government does not take adequate measures to provide remedial classes for the students unable to learn during school closures, learning inequalities may continue to prevail within Nigeria's education sector. Another implication of our findings is that supporting teachers and parents to be able to teach remotely is essential. Although schools have resumed physically, lessons from the pandemic need to be considered for education planning for the future of education in Nigeria.

One of the limitations of this study is that it only covered the first few months of school closure, so we cannot say how and to what extent teaching and learning evolved by the end of the lockdown and school closures. Also, we cannot say anything about the very low-cost private schools that were not able to implement any form of teaching during the period of lockdown, as there is evidence that some students were unable to participate in learning via their schools [6,9,11]. However, as the government programmes were made publicly available, this likely offered alternative learning platforms for students whose schools could not provide any teaching. Another limitation of this study is that we did not examine the quality of the content of the online learning delivered during the pandemic across various school types. Therefore, implications for further research are clear; more research is needed to understand how much children learned and the remedial programmes necessary to bridge any gaps caused by unequal access to education during the lockdown period. Additionally, future studies can explore the long-term impact of COVID-19 on quality and equitable education and the challenges of making digital learning accessible to all learners.

In sum, the advent of the COVID-19 pandemic has presented disruptions to education systems that we are yet to fully understand the impact of, even in the pursuit of the SDG 4 by 2030. As the world continues to grapple with the many consequences of COVID-19 on global education systems, it is crucial to address the deficiencies within teacher education to prepare teachers for a pandemic. Teachers are vital to the success of any education system, and there is a need for the Ministries of Education to address the challenges affecting teachers in their contributions to equitable and quality education for all learners.

## Author contribution statement

Seun Bunmi Adebayo; Gbenga Quadri; Samuel Igah; Obiageri Bridget Azubuike: Conceived and designed the experiments; Performed the experiments; Analysed and interpreted the data; Contributed reagents, materials, analysis tools or data; Wrote the paper.

## Funding statement

The data from this study is from a survey conducted by The Education Partnership (TEP) Centre and the Nigerian Economic Summit Group (NESG), which was supported by Hewlett foundation.

## Data availability statement

Data will be made available on request.

## Declaration of interest’s statement

The authors declare no competing interests.

## Figures and Tables

**Table:** 

School Type	Gender (N)			Age (Mean & SD)		Teaching experience		
	Female	Male	Total	Mean	Standard Deviation	Less than 5 years	More than 5 years	Total
Private School	116	86	202	32.49	7.86	78	124	202
Public School	131	106	237	42.25	10.58	44	193	237
**Total**	**247**	**192**	**439**	**37.76**	**10.6**	**122**	**317**	**439**

## Appendix

Table A1: Descriptive Statistics of Sample.

## Footnotes

1 The Education Partnership (TEP) Centre and The Nigerian Economic Summit Group (NESG), with support from Hewlett Foundation, drew on their competencies in education and economic research to study and report education stakeholders' responses to learning in the wake of the COVID-19 pandemic in Nigeria. www.tepcentre.com.

2 Digital class.
